# Core histone genes of *Giardia intestinalis*: genomic organization, promoter structure, and expression

**DOI:** 10.1186/1471-2199-8-26

**Published:** 2007-04-10

**Authors:** Janet Yee, Anita Tang, Wei-Ling Lau, Heather Ritter, Dewald Delport, Melissa Page, Rodney D Adam, Miklós Müller, Gang Wu

**Affiliations:** 1 Departments of Biology and Chemistry, Biochemistry Program, Trent University, Peterborough, Ontario, K9J 7B8, Canada; 2 Departments of Immunobiology and Medicine, University of Arizona College of Medicine, Tucson, AZ 85724, USA; 3 The Rockefeller University, 1230 York Avenue, New York, NY 10021, USA; 4 Collegium Budapest, H 1012 Budapest, Hungary; 5 Haskins Laboratories and Department of Chemistry and Physical Sciences, Pace University, 41 Park Row, New York, NY 10038, USA

## Abstract

**Background:**

*Giardia intestinalis *is a protist found in freshwaters worldwide, and is the most common cause of parasitic diarrhea in humans. The phylogenetic position of this parasite is still much debated. Histones are small, highly conserved proteins that associate tightly with DNA to form chromatin within the nucleus. There are two classes of core histone genes in higher eukaryotes: DNA replication-independent histones and DNA replication-dependent ones.

**Results:**

We identified two copies each of the core histone H2a, H2b and H3 genes, and three copies of the H4 gene, at separate locations on chromosomes 3, 4 and 5 within the genome of *Giardia intestinalis*, but no gene encoding a H1 linker histone could be recognized. The copies of each gene share extensive DNA sequence identities throughout their coding and 5' noncoding regions, which suggests these copies have arisen from relatively recent gene duplications or gene conversions. The transcription start sites are at triplet A sequences 1–27 nucleotides upstream of the translation start codon for each gene. We determined that a 50 bp region upstream from the start of the histone H4 coding region is the minimal promoter, and a highly conserved 15 bp sequence called the histone motif (*him*) is essential for its activity. The *Giardia *core histone genes are constitutively expressed at approximately equivalent levels and their mRNAs are polyadenylated. Competition gel-shift experiments suggest that a factor within the protein complex that binds *him *may also be a part of the protein complexes that bind other promoter elements described previously in *Giardia*.

**Conclusion:**

In contrast to other eukaryotes, the *Giardia *genome has only a single class of core histone genes that encode replication-independent histones. Our inability to locate a gene encoding the linker histone H1 leads us to speculate that the H1 protein may not be required for the compaction of *Giardia*'s small and gene-rich genome.

## Background

*Giardia *species are binucleated parasitic protists of the group diplomonads [1]. Members of this group differ extensively in their cytology and biochemistry from most other eukaryotes. *G. intestinalis *is the best explored species of this group [2], and its small genome of 12 Mb containing >9,000 ORFs has been completely sequenced recently [3]. As an important human pathogen of the upper intestinal tract, it was in the center of attention of medical parasitologists for many years [4]. Its peculiar cytology, most notably the absence of typical mitochondria, made it an important organism in studies on the evolution of eukaryotic cells [5-8]. During the 1980s and 1990s, these apparently ancestral features led to the assumption that *Giardia *species were representatives of the earliest, premitochondrial branches of the eukaryotic phylogenetic tree. Recent work, however, cast serious doubt on this assessment of diplomonads and suggests that they are derived forms. The presence of mitosomes, organelles that probably derived from the ancestral mitochondrial endosymbiont [9], and mitochondrial genes in its nuclear genome [10,11] indicate that *Giardia *is not ancestrally amitochondriate. Efforts to establish the large scale phylogeny of eukaryotes did not resolve the order of separation of the major eukaryotic lineages and gave no support for an early divergence of diplomonads from other eukaryotes [12].

A tight association between DNA and small, highly basic proteins forms a highly organized arrangement called chromatin in the nucleus. Histones are a major component of these basic proteins, and can be categorized as either core or linker types. In the primary "beads on a string" structure of chromatin, DNA is wrapped around nucleosomes, which are composed of two molecules of each core histone protein (H2a, H2b, H3 and H4). The linker histone H1 binds to the DNA that stretches between adjacent nucleosomes and plays an important role in the formation of higher order chromatin structures within the nucleus. In addition to their role in the packaging of DNA, the core histones can regulate transcription through modifications of their N-terminal tails, which is one of the major mechanisms for epigenetic regulation of gene expression [13]. There are two classes of core histone genes in higher eukaryotes: DNA replication-independent or basal histones, and DNA replication-dependent histones. The genes for the DNA replication-independent histones are dispersed throughout the genome, are constitutively expressed, and their mRNAs are polyadenylated. In contrast, the replication-dependent histone genes are arranged in tandem repeats within the genome, have elevated expression during the S-phase of the cell cycle, and their mRNAs contain a conserved hairpin structure in the 3'UTR instead of a poly(A) tail [14].

In this study, we have characterized the core histone genes of *G. intestinalis*. We determined the copy number, chromosomal localization, and the site of transcription initiation of these genes. The minimal promoter for the histone H4 gene was defined, and the role of a 15 bp conserved sequence within this promoter was analyzed. Furthermore, the binding of proteins from a *Giardia *nuclear extract to the 15 bp motif in the histone H4 promoter was examined. We were unable to detect a gene encoding the linker histone H1.

## Results

Two copies each of the H2a, H2b, and H3 genes, and three copies of the H4 gene were identified in the recently completed *G. intestinalis *genome [3] (Table 1). Surprisingly, no gene encoding a H1 histone was found despite extensive searches. Southern hybridization of BAC clones containing these genes to blots of *G. intestinalis *chromosomes separated by pulsed-field gel electrophoresis showed that the histone genes were dispersed on different chromosomes as follows: one copy of the H2a gene on chromosome 3 and the second copy on chromosome 5; both copies of H2b genes on chromosome 5 separated by approximately 5.8 kb; the two copies of H3 genes on chromosome 4 separated by approximately 0.5 Mb; and the three copies of the H4 genes are localized to a 8 kb fragment near one end of chromosome 5. Two of the three copies of the H4 genes are positioned tail to tail near the end of one contig and the other is at the end of another adjacent contig. This raises the possibility that the detection of three copies of the H4 gene is an artifact of contig assembly and that there are only two copies of the H4 gene as is the case for other *G. intestinalis *core histone genes. To investigate this possibility, we designed PCR primers that flank the genomic DNA region containing the copies of the H4 gene based on the sequence from the *Giardia*DB assembly. Since the DNA fragment obtained from the PCR amplification of *Giardia *genomic DNA with these primers was of the same 8 kb size as the region predicted by the *Giardia*DB assembly (data not shown), the contig assembly of this region is probably correct and three copies of the H4 gene are present.

The DNA sequences within the coding regions were identical for all copies of each *G. intestinalis *core histone gene except for 7 silent nucleotide substitutions between the two copies of the H2a gene. Interestingly, the DNA sequence conservation among the copies of each histone gene continued upstream of the coding region; the length of identical 5' noncoding sequence ranged from 50 bp for the H3 genes to 99 bp for the H4 genes. The lengths of identical 3' noncoding sequence between the copies of each histone gene are much shorter, ranging from only 5 bp to 16 bp, except for the H4 genes, which have 124 bp of identical 3' noncoding sequence. Sequences matching the consensus polyadenylation signal (AGT [A/G]AA [T/C]) of *G. intestinalis *genes [15] were present in the 3' noncoding sequences of all histone genes. This motif overlapped the translation stop codon in both copies of the H3 and H2b genes and in the other histone genes was located at positions from 5 to 20 bp downstream from the stop codon. Examination of 100 bp centered on the putative polyadenylation signal of the genes did not reveal any sequence that could form the stem-loop structure that is conserved in the 3' UTR of mRNAs of replication-dependent histones found in higher eukaryotes [16].

**Figure 1:**
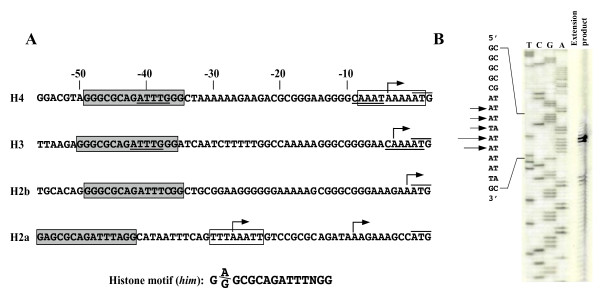
**Determination of the transcription start sites for the *G. intestinalis *core histone genes**. ***A***, 5'RACE analysis of the four core histone genes. The numbers at the top indicate the nucleotide positions in the DNA sequences relative to the translation start codon (*overlined*) for each gene. The *him *sequences are indicated in the *grey boxes; *the AT-rich sequences are indicated in the *open boxes*; the g-CAB elements are *underlined*, and the *bent arrows *indicate the start of transcription for each gene. ***B***, Primer extension analysis of the H4 gene. The extension products were generated from 5'end-labeled H4/PE-1b oligonucleotide and total *Giardia *RNA, and the DNA sequence ladder was generated using the same end-labeled oligonucleotide as a primer. The positions of the major and minor extension products are indicated by *arrows *in the (+)DNA strand shown on the left.

A number of conserved DNA elements were detected in the upstream sequences of the four histone genes (Fig. 1A). The 15 bp conserved motif (G [G/A]GCGCATGATTTNGG) [17], named here as the *hi*stone *m*otif (*him*), is found in every copy of the core histone genes. Two promoter elements, which were first characterized in the glutamate dehydrogenase gene of *G. intestinalis *[18,19], were also recognized in this alignment: the AT-rich sequence was present in the upstream sequence of H4 and H2a, and the *Giardia *CAT box (g-CAB) was present in the upstream sequence of H4 and H3. The transcription initiation site for all four core histone genes was established at triplet A sites near the start of the coding region and downstream from *him *by determining the 5' ends of the histone mRNAs with the technique of rapid amplification of 5' cDNA ends (5' RACE) (Fig. 1A). The start of transcription for the H4 gene was confirmed by primer extension analysis, which produced a major extension product that mapped to the same nucleotide identified by 5' RACE (Fig. 1B). The core histone mRNAs have very short 5' UTRs, ranging from 27 nucleotides for H2a to just a single nucleotide for H2b, which is consistent with reported results for other *Giardia *genes [15].

**Figure 2:**
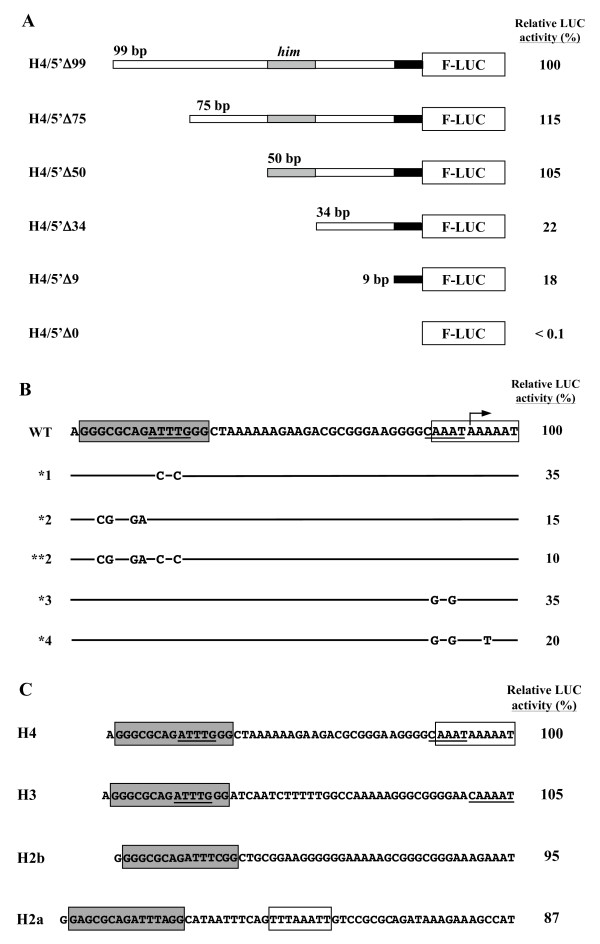
**Promoter analysis of the *G. Intestinalis *core histone genes**. Luciferase activities were determined from *Giardia *transfections with a dual-luciferase reporter system. Each *Giardia *sample was co-transfected with an experimental plasmid and the control plasmid, and assayed sequentially for firefly and *Renilla *luciferase activities. The firefly luciferase activity was divided by the *Renilla *luciferase activity to obtain the F/R-LUC ratio. Percentages of relative luciferase activity were calculated by comparing the F/R-LUC ratio obtained from *Giardia *transfected with each construct relative to the ratio obtained upon transfection of the control plasmid in each experiment. ***A***, Identification of the histone H4 promoter. Experimental plasmids contained incremental deletions of the upstream region of the H4 gene to drive the expression of the firefly luciferase (F-LUC) gene. The composition of the experimental constructs are represented by: *white bar*, 5' noncoding region of the H4 gene; *grey bar*, *him *sequence; *black bar*, AT-rich sequence; *open box*, firefly luciferase coding region. The numbers proximal to the *white bars *indicate the length of 5' noncoding region of the H4 gene remaining within each plasmid. ***B***, Mutational analysis of the histone H4 promoter. Experimental plasmids contained mutations within the 50 bp promoter region of the histone H4 gene to drive the expression of the firefly luciferase (F-LUC) gene. In the wild-type H4 promoter sequence presented on the top line, the *him *is indicated by the *grey box*; the AT-rich sequence is indicated by the *open box*; the g-CAB elements are underlined; and the transcriptional start site is indicated by the *bent arrow*. ***C***, Comparison of the four core histone promoters. The minimal 5' noncoding sequence of each core histone gene that contain the *him *sequence was used to drive the expression of the firefly luciferase gene in the experimental plasmid constructs. The *him *sequences are indicated by *grey boxes*; the AT-rich sequences are indicated by *open boxes; *and the g-CAB elements are *underlined*.

To study the role of *him *in the expression of the core histone genes, we used the upstream sequence of the H4 gene to drive the expression of the luciferase reporter gene in transient transfections of *Giardia *(Fig. 2A). An initial 99 bp of H4 upstream sequence was used because this is the length of sequence that is identical between the two copies of the H4 gene. Only minor effects on luciferase activity were observed when the 5' flanking sequence was deleted to within 50 bp of the translation start codon (H4/5'Δ50). However, luciferase activity decreased five-fold if the *him *sequence was removed (H4/5'Δ34 and H4/5'Δ9), and dropped to background levels when the remainder of the 5' flanking sequence was eliminated (H4/5'Δ0).

To further test the function of the conserved motifs in the histone H4 promoter, we made mutations to the 50 bp upstream sequence contained in the plasmid H4/5'Δ50 and assayed their effects on luciferase activity in *Giardia *transfections (Fig. 2B). Mutations to the g-CAB element within *him *(*1), as well as mutations in *him *outside of the g-CAB element (*2) caused marked decreases in luciferase activity. Combining the *1 and *2 mutations of *him *into a single plasmid (**2) resulted in a further decrease in luciferase activity. Reductions in luciferase activities were also observed when mutations were introduced into the g-CAB element overlapping the AT-rich sequence (*3) and in the AT-rich sequence itself (*4).

The relative strengths of the core histone promoters were tested by using the minimal upstream sequence of each gene that contained the *him *sequence to drive the expression of luciferase reporter constructs (Fig. 2C). Transfections of these constructs into *G. intestinalis *showed that all four core histone promoters have similar activities. The relative expression of the four histone genes was nearly equal as shown by similar C_t _(cycle threshold) values obtained in real-time qPCR experiments (Table 2). By comparison, similar analysis of mRNA for 11 other protein-encoding genes in *G. intestinalis *gave C_t _values that differ as much as 7 to 9 cycles (J. Yee, unpublished).

We also found that the relative mRNA levels of the four core histone genes did not differ in cells from exponential phase or from stationary phase cultures. The RNA was extracted from these cultures and cDNAs were synthesized using either a poly(T)_25 _primer or a primer specific for each histone gene. The cDNAs were then used as templates in PCR reactions on a real-time PCR instrument. Table 2 shows the qPCR results from cDNA synthesized from poly(T)_25_-primed RNA extracted from exponential cultures. The average C_t _numbers obtained for the four core histone genes differ by less than 2 amplification cycles; the numbers for the H4 and H3 genes are nearly identical, while those for H2a and H2b genes are slightly higher. Similar results were obtained when these analyses were repeated with cDNA generated from exponential phase RNA by gene specific primers, and with cDNA generated either by poly(T)_25 _or gene specific primers from RNA extracted from stationary phase cultures (data not shown).

**Figure 3:**
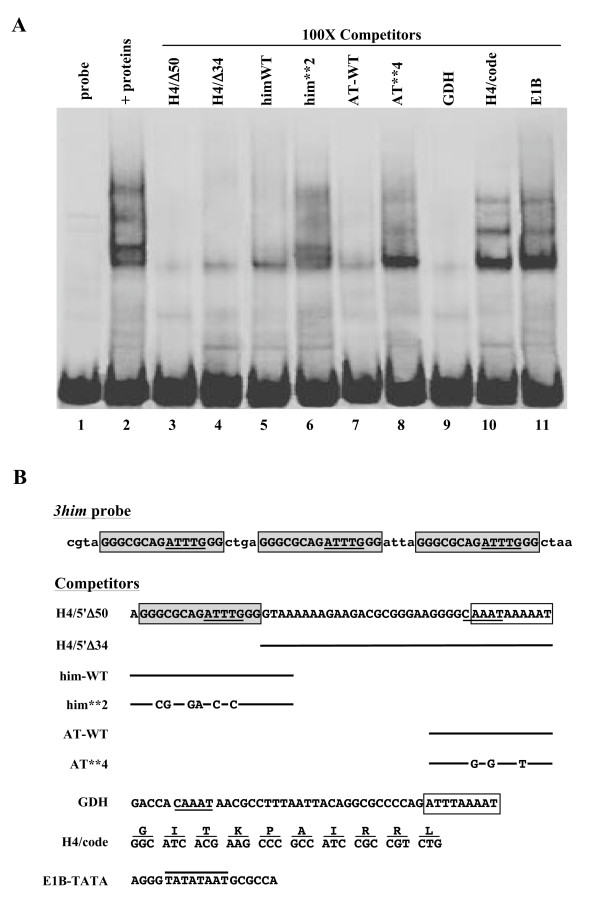
**Characterization of protein binding to the *him *sequence**. ***A***,Gel-shift assays were performed with 5' biotin-labeled, double-stranded DNA containing three histone motifs in tandem (*3him*) and crude nuclear extract from *Giardia*. Lane 1 contains the probe alone; lane 2 contains the probe plus nuclear proteins; lanes 3–11 contain unlabeled DNA used at 100X molar excess relative to the probe. ***B***, The probe and competitors were generated by annealing complementary oligonucleotides together, although only the sequences of the plus strands are shown. DNA motifs within the sequences are indicated: *grey box *for *him*, *open box *for the AT-rich element; g-CAB elements are *underlined*; and the TATA-box of the E1B adenovirus is *overlined*.

To test whether the *him *sequence is a binding site for transcription factors, gel-shift assays were performed with the use of a double-stranded DNA probe containing three *him *motifs in tandem (*3him*, Fig. 3). Several shifted complexes were observed when this probe was incubated with proteins from a *Giardia *nuclear extract (Fig. 3A). The DNA sequence specificity of the binding was tested by the addition of unlabeled, double-stranded DNA containing different sequences at 100-fold molar excess to the probe. Unlabeled DNA containing the 50 bp minimal histone H4 promoter (H4/5'Δ50) or containing a single *him *sequence (*him*WT) were able to compete for protein binding to the probe (lanes 3 and 5 in Fig. 3A). However, if him**2 DNA with the mutations that cause a reduction in luciferase activity in transfections (**2 in Fig. 2B) was used as competitor, it had much less effect on protein binding (lane 6 in Fig. 3A). DNA containing a sequence from the coding region of the histone H4 gene (H4 code) did not compete with the 3*him *probe, nor did DNA containing the adenovirus E1B TATA-box (E1B). Unexpectedly, the H4 promoter with deleted *him *sequence (H4/5'Δ34), and the minimal promoter of the *G. intestinalis *glutamate dehydrogenase (GDH) gene that contains no *him *sequence were also able to compete for protein binding (lanes 4 and 9 in Fig. 3A). To investigate the possibility that the AT-rich element present in H4/5'Δ34 and the GDH promoter is competing with protein binding to *him*, DNA containing a single AT-rich element (AT-WT) was used as a competitor in the gel-shift assay. The AT-WT DNA was able to compete with the 3*him *probe for protein binding (lane 7 in Fig. 3A), but an excess of AT*4 (lane 8 in Fig. 3A), which contained mutations in the AT-rich element that caused a reduction in luciferase activity in *Giardia *transfections (*4 in Fig. 2B), was greatly reduced in its ability to compete with the 3*him *probe.

## Discussion

The sequences of the four core histones (H2a, H2b, H3 and H4) of *Giardia intestinalis *are similar to those of histones from other eukaryotes [17]. Protein structure modeling of the putative translation products indicate that these proteins can assemble into nucleosomes that do not differ significantly from nucleosomes from vertebrates [17]. In the present study we extend this information to the copy number, promoter structure, and genomic distribution of the histone genes in this organism. Each of these genes is represented in its genome by two copies only – with the exception of the H4 gene that is present in three copies. These copy numbers are at the low end of the scale observed among eukaryotes, ranging from just two copies of each gene in yeast, 10 to 20 copies in humans, and up to several hundred copies in sea urchins [20]. The unexpected extensive DNA sequence identities between the copies of each of these genes, especially since they extend to the 5' noncoding region, suggest that these copies have arisen from relatively recent gene duplications or gene conversions.

All canonical replication-dependent histone genes in higher eukaryotes lack polyadenylation signals except for some invertebrate animals (insects [21,22], worms [23], crustaceans [24], and mussels [25]) that have core histone genes with both a stem-loop structure and a polyadenylation signal. These findings have led to the hypothesis that there has been a progressive replacement of the polyadenylation signal by the stem-loop structure in replication-dependent histones in defining the 3' ends of core histone mRNAs during animal evolution [25]. The presence of polyadenylation signals and the absence of potential stem-loop structures in the 3' noncoding sequences of the *Giardia *core histone genes suggest that studies on representatives of other eukaryotic lineages is warranted to determine whether a similar evolutionary trend is a more general phenomenon.

The linker histone H1 is a ubiquitous component of eukaryotic chromatin and is expected to be present in this protist. An earlier report putatively identified a 21 kDa basic protein from nuclear extracts as histone H1 [26]. The identification was based on the size of the protein and on the sequence of one of its peptides (VAATPVSTKAAP) that was highly similar to a sequence within the chicken histone H1 protein (VAAPPTPAKAAP). Our inability to find this peptide or any homologs of H1 sequences from other organisms in the *G. intestinalis *genome database is therefore puzzling. More than an 11-fold coverage of the genome has been achieved by the *Giardia *genome sequencing project [3], so the possibility that the H1 gene was missed in the assembly is unlikely although it cannot be excluded. Furthermore, we were not able to detect specific binding to any protein in a *Giardia *histone extract with an antibody that recognizes the H1 protein in chicken, human and other mammals in Western blot analysis (data not shown).

These observations raise the problem of the identity of the protein isolated by Triana *et al*. [26]. The failure to identify a histone H1 in the *Giardia *genome indicates that this gene is either so divergent that it could not be recognized by our database searches or that it absent from this organism. No unambiguous H1 has been reported for Apicomplexa [27], and the H1 gene is non-essential in the ciliate, *Tetrahymena thermophila *[28]. Although TEM analysis of *Giardia *chromatin by Triana *et al*. showed structures that were more compact than the "beads-on-a-string" nucleosome filament, it is unclear whether a 30 nm fibre structure is present. Indeed, polynucleosome fibres in the presence of physiological levels of cations can fold further to form more compact chromatin structures in the absence of H1 histones [29,30]. In *Giardia*, more than 9,000 ORFs are crammed into a genome that is only 12 Mb in size. Thus, it is tantalizing to speculate that the formation of histone H1-dependent chromatin structures may not be necessary to compact such a small and gene-rich genome. In support of this idea, we were also unable to identify a histone H1 in the Microsporidian, *Encephalitozoon cuniculi*, an evolutionarily distant organism with a genome less than 3 Mb in size that contains almost 2,000 protein encoding genes [31]. Only further biochemical studies on *Giardia *chromatin can clarify the conundrum of the missing histone H1 gene. If *Giardia *indeed lacks a histone H1, this finding would be consistent with the hypothesis that H1 histones were recruited in eukaryotic evolution after the acquisition of the core histones to further refine the chromatin structure [32,33]. However, further support for this hypothesis would require more detailed analysis of representative organisms from different lineages.

Whereas the evolutionary origin of the core histones can be traced back to a DNA binding protein in archaebacteria, such as the Hmf protein in *Methanofermus fervidus *[34,35], the origin of the H1 histone is more difficult to determine. Unlike the highly conserved core histone proteins, the H1 proteins are very heterogeneous among protozoa, and exhibit great diversification and specialization even among mammals [32]. Nevertheless, the sequence similarity of small basic proteins found in several eubacteria to the lysine-rich carboxyl terminus of metazoan H1 proteins has led to speculations that these eubacterial proteins are candidates for the ancestral histone H1 protein [36]. In the dinoflagellate, *Crypthecodinium cohnii*, proteins with similarity to the core histones are absent but two proteins (HCC1 and HCC2) with significant similarity to the small molecular weight HU bacterial protein are present [37]. The HU protein is the most abundant DNA-binding protein in *E. coli*, and one role of this multifunctional protein is the organization of the bacterial chromosomal DNA [38,39]. Intriguingly, an HU-like DNA-binding protein was also identified by Triana *et al*. [26] based on a peptide sequence obtained from a small molecular weight protein on a SDS-PAGE gel of fractionated *Giardia *chromatin. However, we were unable to locate an exact match of this peptide sequence in the *Giardia *genome database by BLASTp and tBLASTn searches. The *Giardia *sequence with the best match to the putative HU peptide was only 8 out of 13 amino acids. Negative results were also obtained when the *Giardia *genome was searched with the full-length HU sequence from *Bacillus subtilis*. When the top five *Giardia *sequences from each of the above searches were used individually in BLASTp and tBLASTn searches against all sequences in the GenBank database, no HU nor other histone sequences were retrieved. Possible explanations for our lack of finding the HU gene is that the HU-like gene was missed in sequencing of the *Giardia *genome, or more likely, there was a bacterial contamination of Triana *et al*.'s *Giardia *chromatin preparation.

The transcription initiation site of the *G. intestinalis *histone genes was located only a few nucleotides upstream from the translation start codon, in agreement with previous studies showing that mRNAs of this species have unusually short 5' UTRs [2]. The translation of messages with such short 5' UTRs has been a point of debate because earlier reports suggested that *Giardia *mRNA are uncapped [40]. Recent studies, however, demonstrated more convincingly that *Giardia *mRNAs are capped, and that capped mRNAs with 5'UTRs as short as a single nucleotide can be efficiently translated in this protist [41,42].

Our results obtained with transient transfection assays with luciferase activity as indicator show that an about 50 bp upstream stretch has full promoter activity for *G. intestinalis *core histone genes. This is in agreement with results of transcriptional analysis of other *Giardia *genes demonstrating promoter regions range from 40 to 60 bp in length [18,19,43,44]. Mutational analysis of these regions showed that the promoter elements are only weakly conserved and usually contain triplet A's and/or triplet T's [18,19,43,44]. The 15 bp histone motif (*him*) with the consensus sequence GRGCGCAGATTNGG, was detected in all four core histone genes, but has not been found in other *Giardia *genes or in the core histone genes of other eukaryotes [17]. Motivated by the assumption that *him *is a regulatory element that controls the coordinated expression of the *Giardia *core histone genes, we decided to characterize the promoter of the histone H4 gene by analyzing its upstream sequence in more detail. The marked decrease in luciferase activity observed with deletions or mutations in *him *indicates the importance of this motif to the function of the histone H4 promoter. The highly conserved nature of the motif, and our observation that the four histone promoters had approximately equivalent activity, suggest that *him *also has an important regulatory role in the transcription of the other *Giardia *core histone genes.

We used quantitative real time PCR to compare the steady state mRNA levels of the four histone genes and found that they were all within a four-fold range (two amplification cycles) of each other. We did not detect any significant differences in the relative levels of core histone mRNAs extracted from exponential phase cultures compared to mRNAs from stationary phase cultures. Moreover, we have not observed any marked changes in core histone mRNA levels during the cell cycle in our analysis of gene expression in semi-synchronized *Giardia *cultures (J. Yee, unpublished data). These results suggest that the core histone genes of *G. intestinalis *are constitutively expressed at approximately equivalent levels. These observations are also consistent with the absence of orthologs in this species to the transcription factors SPT10, SPT21, HIR1 or HIR2, which are involved in the expression of replication-dependent histones in yeast [45,46]. Therefore, the *him *sequence is likely to be a binding site for a transcription factor that allows a relatively high and constant level of histone gene expression in *Giardia*. The equivalent results obtained by qRT-PCR assays using cDNA produced from mRNA with either poly(T) or gene-specific primers demonstrate that the histone transcripts are polyadenylated. These observations suggest that the single class of core histone genes in *Giardia *have a dual function: they provide bulk histones for packaging of newly synthesized DNA during S-phase in the cell cycle, and provide replacement histones for the repair of chromatin during the other stages of the cell cycle.

While information on the identity and function of transcription factors in *G. intestinalis *is scarce, a recent survey of its genome identified only four general transcription initiation factors among the twelve that are normally associated with transcription in higher eukaryotes [47]. Furthermore, a *Giardia *TBP was identified that is highly divergent with respect to archaeal and higher eukaryotic TBPs, and it contains substitutions in three out of four phenylalanines required for binding of other TBPs to TATA-sequences [47]. In this study, we showed that proteins from a *Giardia *nuclear extract bound to a probe containing three *him *sequences in tandem, and demonstrated the specificity of these DNA-protein interactions by competition assays with an excess of unlabeled DNA containing different sequences. Competition was observed with unlabeled DNA containing the 50 bp minimal H4 promoter or a single wild type *him *sequence, but not with a region of the H4 coding sequence or with a canonical eukaryotic TATA-box sequence. In addition, a mutated sequence of *him *had greatly reduced ability to compete for protein binding to the probe. While these results suggest that a protein or proteins are binding specifically to *him*, the competition observed with DNA containing the H4 promoter with the *him *deleted and the GDH promoter lacking *him *appears to contradict this conclusion. However, these two sequences contain both an AT-rich and a g-CAB element, and we showed that the wild-type AT-rich sequence was able to compete for protein binding to the 3*him *probe but the mutant AT-rich sequence could not. Taken together, these results suggest that either a common protein is binding to *him *as well as to the g-CAB and AT-rich elements, or more likely, a common cofactor is required for the formation of different protein complexes at each of these promoter elements. One possible candidate for this common cofactor is *pot*, a protein that was described in our previous characterization of the GDH promoter [18,19]. However, the identity of the proteins that bind *him *awaits the completion of our protein purification experiments.

## Conclusion

In summary, we identified a single class of core histone genes in *G. intestinalis *that are constitutively expressed at relatively high levels, and their mRNAs are polyadenylated. There are two copies each of the core histone H2a, H2b and H3 genes, and three copies of the H4 gene, at separate locations on chromosomes 3, 4 and 5 within the genome of *Giardia intestinalis*. The low copy number of each basal histone gene, and the lack of a second class of replication-dependent genes may be further examples of genetic downsizing in this protist. Our inability to locate a gene encoding the linker histone H1 leads us to speculate that the assembly of higher ordered chromatin structures that are H1-dependent may be restricted in *Giardia*. If so, chromatin remodelling in this organism would be more dependent on histone modifications and the substitutions of histone variants into the nucleosomes. We determined that a 50 bp region upstream from the start of the histone H4 coding region is the minimal promoter, and a highly conserved 15 bp sequence motif called the histone motif (*him*) is essential for its activity. Our gel-shift assays showed that a common factor is shared between the protein complex that binds to *him *and the complexes that bind other promoter elements described previously in *Giardia*. Given the short lengths (50 – 60 bp) of promoters in this protist, it is likely that only a single protein complex, composed of a core set of transcription factors, can form on these regions. Gene-specific transcription factors, such as the *him*-binding protein, would interact with and modulate the activity of the core complex. The implications of such genetic streamlining on the nature of transcription initiation in *Giardia *are unknown. Further study of these transcription factors and chromatin structure in *G. intestinalis *would provide a clearer picture of gene expression in this remarkable eukaryote.

## Methods

### Database searches

The programs of BLASTp and tBLASTn were used for sequence search [48]. Nucleotide sequences of the four core histone genes of *G. intestinalis *have been established earlier (NCBI GenBank accession numbers AF139873–AF139876; [16]). These sequences were used as queries to search the public database of the *G. intestinalis *(= *G. lamblia*) genome project [3] for gene copy number and flanking sequences. In order to identify genes encoding linker histone H1, the genomes of *G. intestinalis *[49] and the Microsporidian, *Encephalitozoon cuniculi *[50] were searched using histone H1 sequences  from the NCBI protein database for *Saccharomyces cerevisiae *(accession P53551), *Homo sapiens *(accession CAA40409), *Caenorhabditis elegans *(accession CAA37372) and G*allus gallus *(accession P08285). A partial sequence (VAATPVSTKAAP) from a putative H1, and a partial sequence (TIEIPESNVPAFK) from a putative HU obtained from *G. intestinalis *nuclear extract [25], and the full-length HU sequence from *Bacillus subtilis *(accession P08821) were also used as queries in searches of the *G. intestinalis *genome with a E-value set at 1000 to identify short or divergent sequences. Four *Giardia *sequences with the best matches from each of these searches were retrieved and used as queries in BLAST searches of the GenBank database.

Searches for potential stem-loop structures within the 100 bp region centered on the translation stop codon of each *Giardia *core histone gene were performed using the PrimerSelect module of the Lasergene software (DNASTAR).

### Chromosomal localization of histone genes

As part of the *G. intestinalis *genome project [3], multiple BAC clones containing *G. intestinalis *genomic DNA were sequenced at the ends to allow localization to contigs and supercontigs. The sizes of selected BACS were determined by restriction digestion using rare-cutting enzymes, giving an insert size of 50 to 240 kb. Selected BACs were also hybridized to *Giardia *chromosomes separated by pulsed field gel electrophoresis (PFGE) as described (Adam 1988) to allow chromosomal assignment. In this way, all the larger contigs and supercontigs were assigned to specific chromosomes (Adam, unpublished), and the chromosomal locations of the *G. intestinalis *histone genes were determined.

### 5' RACE

Axenic *G. intestinalis *(strain WB clone 6; ATCC 30957) was cultured in modified TYI-S-33 medium [51]. Total RNA was extracted from trophozoite cultures by the use of the TRIZOL Reagent (Invitrogen). A RLM-5'RACE (RNA ligase-mediated rapid amplification of 5' cDNA ends) reaction was carried out for each core histone gene by using FirstChoice^® ^RLM-RACE Kit (Ambion) with approximately 11 μg of total RNA per reaction. The RNA sample was treated with calf intestine alkaline phosphatase to remove the 5' phosphate group from any degraded RNA and DNA. Tobacco acid pyrophosphatase was added to remove the cap structure from the 5' end of the mRNAs, and an oligonucleotide adapter was ligated to the 5' end of the treated mRNA. The mRNA samples were reverse transcribed from random 10-nucleotide primers to produce the first strand of cDNAs, which were subsequently amplified in two sequential PCR reactions. In the first reaction the outer primer complementary to the anchor and the outer gene-specific primer were used. In the second reaction the inner primer complementary to the anchor and the inner gene-specific primer were used. The outer and inner anchor primers were supplied with the RLM-5'RACE kit. The sequences of the outer and inner gene-specific primer are listed in Table 3. Since only cDNAs that are extended to full-length will have the anchor sequence at their 3' ends, truncated cDNA will not be amplified in these PCR reactions. The amplicons were cloned into a TA plasmid using the TOPO TA Cloning Kit (Invitrogen). Three to five plasmid clones from each RLM-5'RACE reaction were sequenced. The position in the DNA sequence adjacent to the ligated adapter corresponded to the transcription start site.

### Primer extension analysis

The primer extension analysis was performed as described previously [52]. An oligonucleotide, H4/PE-1b (5' GAT GGC GGG CTT CGT GAT GCC 3') with a sequence complementary to codons 25 – 31 of the *G. intestinalis *histone H4 gene was 5' end-radiolabeled with ^33^P using T4 polynucleotide kinase (New England Biolabs) and annealed to 10 μg of *Giardia *total RNA. The primer was extended with SuperScript™ II reverse transcriptase (Invitrogen) and dNTPs (0.67 mM). The RNA in the sample was degraded by incubating it with 0.5μl of DNase-free RNase A (Promega) for 15 minutes at 37°C. The remaining cDNAs in the sample were electrophoresed on a denaturing 6% PAGE. A sequence ladder was generated with the use of the same end-labeled oligonucleotide as a primer in a DNA sequencing reaction with a plasmid containing the *Giardia *histone H4 gene as the template.

### Plasmid construction

The transfection plasmids were constructed starting from the plasmids, GDH/5'Δ5N and pRL-null. The GDH/5'Δ5N plasmid contains the firefly luciferase gene flanked by 44 bp of upstream sequence and 120 bp of downstream sequence from the *G. intestinalis *glutamate dehydrogenase gene as described previously [19]. The pRL-null plasmid (Promega) was the source of the *Renilla *luciferase gene.

The control plasmid, R-Luc, was constructed by replacing the firefly luciferase gene in GDH/5'Δ5N with the gene for *Renilla *luciferase from pRL-null.

The GDH/5'Δ5N plasmid was digested with *Hin*dIII and *Nco*I to generate a 44 bp fragment containing the GDH minimal promoter and a 4,964 bp fragment containing the promoterless vector we called NΔ2N. The "deletion" constructs shown in Fig. 2A were made by inserting DNA fragments containing varying lengths of the upstream sequence from the histone H4 gene into the linearized NΔ2N vector. The 99 bp and 75 bp of H4 upstream sequences, contained in H4/5'Δ99 and Δ75, respectively, were generated by PCR amplification of *G. intestinalis *genomic DNA with forward primers containing *Hin*dIII sites at their 5' ends, and reverse primers containing *Nco*I sites at their 5' ends. The 50 bp, 34 bp and 9 bp of H4 upstream sequences, contained in H4/5'Δ50, Δ34 and Δ9, respectively, were obtained by annealing complementary oligonucleotides that left overhanging *Hin*dIII and *Nco*I sites at the 5' and 3' ends, respectively.

The mutant constructs shown in Fig. 2B were made by replacing the 50 bp promoter of histone H4 H4/5'Δ50 with duplex oligonucleotides containing nucleotide substitutions within its sequence. The constructs shown in Fig. 2C containing the putative promoters for the H3, H2b and H2a genes were made by inserting duplex oligonucleotides containing the respective sequences into the NΔ2N vector. All constructs were checked by DNA sequencing.

### *Giardia *transfections and luciferase assays

The Dual-Luciferase^® ^Reporter Assay System (Promega) was utilized in transfections to study the core histone promoters. The experimental reporter was the firefly luciferase (F-Luc) gene driven by different sequences upstream of the *Giardia *core histone genes, and the control reporter was the *Renilla *luciferase (R-Luc) gene driven by the minimal promoter of the *Giardia *glutamate dehydrogenase (GDH) gene [18,19].

*G. intestinalis *was grown and prepared for electroporation as described previously [53]. The test F-Luc plasmid DNA (40 μg) and the control R-Luc plasmid DNA (5 μg) were added to the cells immediately before electroporation. The *Giardia *cells were placed back into culture, and collected after a 6 h recovery at 37°C as previously described [53]. The cells were lysed by resuspension in 25 or 30 μl of passive lysis buffer (Promega) supplemented with 0.75 μg/ml leupeptin (Sigma), and one freeze/thaw cycle. For each dual luciferase assay, 20 μl of the *Giardia *lysate was mixed with 100 μl of luciferase assay reagent II (Promega), and placed in the luminometer (Turner Designs TD-20/20 Luminometer) to measure the firefly luciferase (F-Luc) activity. To stop the F-Luc reaction and provide the substrate for the *Renilla *luciferase (R-Luc), 100 μl of Stop & Glo reagent (Promega) was added to the sample tube, vortexed briefly, and placed back in the luminometer to measure the R-Luc. The F-Luc/R-Luc ratio was calculated by dividing the F-Luc value by the R-Luc value for each sample.

In every experiment, each electroporation was performed in triplicate. The relative luciferase activities listed under "Results" (as percentages of the F-Luc/R-Luc ratios obtained in control transfections) are averages of three to six independent experiments and have standard deviations within 10% of the presented value.

### Real-time RT-PCR

The sequences of the primers used in the real time quantitative RT-PCR (RT-qPCR) reactions are shown in Table 2. StrataScript RT enzyme (Stratagene) was used to synthesize first strand cDNAs from either an oligo d(T)_21 _or a gene-specific primer annealed to the mRNAs. For each gene, PCR reactions with cDNAs synthesized from RNA concentrations at 50, 25, 10, 5, 1 and 0.5 ng/μl were performed. Two control reactions were also performed for each gene: an RNA sample from a mock cDNA synthesis reaction containing no reverse transcriptase (No-RT), and a control sample without template (NTC). In the No-RT sample, RNA was used at 50 ng/μl instead of cDNA; in the NTC sample, water was added instead of the cDNA template.

Each 25 μl PCR reaction contained: 9.13 μl of nuclease-free water, 2.5 μl of 10X PCR buffer (200 mM Tris-HCl, pH 8.4, 500 mM KCl), 2.5 μl of 25 mM MgCl_2_, 2.0 μl of dNTP mixture (containing 5 mM of each dNTP), 0.75 μl of DMSO, 4 μl of 50% glycerol, 0.37 μl ROX reference dye (Stratagene), 1.25 μl of 3.3X SYBR green (Cambrex), and 0.5 μl of Taq DNA polymerase (Invitrogen). The real-time PCR reactions were performed on the Mx3000P instrument (Stratagene) as follows: one cycle at 95°C for 10 min; 40 cycles at 94°C for 30 seconds, 60°C for 1 minute, and 72°C for 30 seconds. Fluorescence levels were measured during the 60°C annealing phase of each cycle as well as continuously during the dissociation analysis.

A standard curve was constructed for every gene, and the efficiency of PCR amplification was calculated from the slope of the plot (% efficiency = [10^-1/slope ^- 1] * 100). After the PCR reaction, a melting curve analysis of the amplified product was performed for each gene to detect the presence of any primer dimers. Each standard reaction was performed in triplicate. All reactions were performed on the same 96-well plate in each experiment, and each experiment was performed at least three times.

### Gel-shift assays

*G. intestinalis *nuclear extracts were prepared as described previously [19]. The double-stranded DNA probe was prepared by heating equimolar amounts of complementary 5' biotin-labeled oligonucleotides containing three *him *sequences in tandem at 90°C for 10 minutes in annealing buffer (5 mM Tris-HCl, pH 7.9, 1 mM MgCl_2_, 10 mM NaCl, 0.1 mM DTT), and then allowing the oligonucleotides to anneal slowly as the sample cooled to room temperature. Double-stranded DNA competitors were prepared in the same manner as the probe except these complementary oligonucleotides were unlabeled (see Fig. 3B for the sequences of the probe and competitors).

A typical binding reaction contained 1–2 pmole of probe, 7–15 μg of nuclear extract in 1X binding buffer (10 mM Tris-HCl, pH 7.5, 50 mM NaCl, 0.5 mM DTT and 5% glycerol) plus 0.2 μg poly(dI/dC) and 0.4 μg leupeptin. In competition experiments, all components, including the probe and competitor DNA, were pre-mixed before the addition of protein extract. Reaction mixtures were incubated for 15 minutes at room temperature and resolved on a pre-electrophoresed 5% native polyacrylamide gel.

After electrophoresis the DNA in the gel was transferred onto a 0.45 μM nylon membrane (MagnaProbe^®^) in a semi-dry electroblotter at 200 V for 30 minutes. The DNA was UV crosslinked onto the membrane by placing the membrane on a transilluminator for 3 min. The membrane was incubated in 20 ml of 1X blocking buffer (0.1 M Tris-HCl, pH 7.5, 0.15 M NaCl, 0.1% skim milk powder) for 30–60 minutes at room temperature or overnight at 4°C. Next, the membrane was incubated for 30 minutes in 20 ml of 1X blocking buffer with 2 μl of streptavidin-AP antibody (Roche) added to obtain a 1/10000 dilution of the antibody. The membrane was incubated twice for 15 minutes in washing buffer (100 mM maleic acid, 150 mM NaCl, and 0.3% Tween-20), and then equilibrated in detection buffer (0.1 M Tris-HCl, 0.1 M NaCl, pH 9.5) for five minutes. The membrane was removed from the detection buffer and 6–8 drops of the CDP-star chemiluminescent reagent (Perkin-Elmer) were applied to the membrane. After a pre-incubation period at 37°C for 5 minutes to enhance the chemiluminescence signal, the membrane was placed into the cabinet of a Chemigenius^2 ^bioimaging system (Syngene) to visualize the biotin-labeled DNA bands.

## List of abbreviations

3'UTR : 3' untranslated region of the mRNA

5'RACE : Rapid PCR amplification of the 5' cDNA ends

5'UTR : 5' untranslated region of the mRNA

AT-rich sequence : A conserved AT-rich sequence containing the transcription initiation site found in many *Giardia *gene promoters

BAC : Bacterial artificial chromosome

C_t _: Cycle threshold; the number of PCR cycles required in a real-time PCR reaction to reach a set fluorescent threshold

F-Luc : Firefly luciferase

g-CAB : *Giardia *CAT box promoter motif

GDH : Glutamate dehydrogenase

*him : *A 15 bp conserved motif in the promoters of the *Giardia *core histone genes

*HIR1 *and *HIR2 : *Transcriptional corepressors involved in the cell cycle-regulated transcription of core histone genes in *Saccharomyces cerevisiae*

No-RT : A negative control sample for real-time RT-PCR experiments where no reverse transcriptase is added to the cDNA synthesis step

NTC : A negative control sample for real-time PCR experiments where no template (no RNA nor DNA) is added to the PCR reaction

ORF : Open reading frame

PFGE : Pulsed field gel electrophoresis

*pot *: Poly(T)-binding protein

qPCR : Quantitative real-time PCR

qRT-PCR : Quantitative real-time RT-PCR

RLM-5'RACE : RNA ligase-mediated rapid amplification of 5' cDNA ends

R-Luc : *Renilla *luciferase

S-phase:  DNA synthesis phase of the cell cycle

*SPT10 *and *SPT21 *: Sequence-specific activator of core histone genes in *Saccharomyces cerevisiae*

TBP : TATA-binding protein

TEM : Transmission electron microscopy

## Authors' contributions

JY: Principal Investigator of the research described; designed the experiments; performed a portion of the *Giardia *transfection experiments; analyzed and interpreted the results; wrote and revised the manuscript.

AT*: Undergraduate Student; performed the 5' RACE experiments on the histone H2a, H2b, and H3 genes; performed a portion of the qPCR experiments.

WLL*: Undergraduate Student; performed the *Giardia *transfections and deletion analysis of the histone H4 gene; performed the primer extension analysis of the histone H4 gene.

HR*: Undergraduate Student; performed the 5' RACE analysis on the histone H4 gene; performed a portion of the qPCR experiments.

DD*: Undergraduate Student; performed the *Giardia *transfections and mutational analysis of the histone H4 gene.

MP*: M.Sc. Student; performed the gel-shift assays to characterize the protein binding to the *him *sequence

RA: Collaborator; provided the results of the BAC hybridization for the chromosomal localization of the core histone genes; provided critical revision of the manuscript.

MM and GW: Collaborators; first to discover the histone motif in the upstream region of the core histone genes; provided the genomic clones of the core histone genes and WB *Giardia *isolate; performed the bioinformatic searches; discussion of the results; provided critical revision of the manuscript.

All authors read and approved the final manuscript.

*Students supervised by Janet Yee at Trent University

## Figures and Tables

**Table 1:** Copy number and genomic organization of core histone genes

		**Chromosome**^**2**^				**Poly(A)**				**Conserved sequences**^**5**^		
**Histone**	**ORF ID**^**1**^	**#**	**Position**	**Dir.**		**Sequence**^**3**^	**STOP**^4^			**Upstream**	**Coding**	**Downstream**

**H4-i**	135001	5	0.6	R		**AGTAAAC**	-5 bp			99 bp	300 bp	124 bp
**H4-ii**	135002	5	0.6	F								
**H4-iii**	135003	5	0.6	R								

**H3-i**	135231	4	0.2	R		**AGTAAAC**	overlap			50 bp	441 bp	5 bp
**H3-ii**	14212	4	0.7	R								

**H2a-i**	27521	3	0.15	F		**TGTAAAC**	-10 bp			80 bp (-2)	375 bp (-7)	12 bp (-1)
**H2a-ii**	14256	5	0.6	R		**TGTGAAC**	-20 bp					

**H2b-i**	121046	5	1.2	R		**AGTAAAT**	overlap			56 bp	393 bp	16 bp
**H2b-ii**	121045	5	1.2	R								

^1 ^ORF ID as assigned for each gene on *Giardia*DB [3].

^2 ^The chromosome position indicates the distance in Mb from the end of the map for that chromosome. The distance from the telomere is greater, since the map does not extend all the way to the telomeres.

^3 ^Sequence matching the consensus polyadenylation signal for *Giardia *genes (Reviewed in [15]); the DNA sequence for the stop codon is underlined in the polyadenylation sequence shown for the H2B and H3 genes since these sequences overlap for these two genes.

^4 ^Location of putative polyadenylation signal relative to the translation stop codon of each gene.

^5 ^Coding and non-coding sequences conserved among all copies of each core histone gene; number of nucleotide mismatches are indicated in brackets.

**Table 2:** Relative expression of *Giardia intestinalis *core histone genes

**Gene**	**Accession #**^**1**^	**PCR primers (5' → 3')**^**2**^	**Avg. PCR efficiency**^**3**^	**Avg. C**_**t**_^**4**^
**H4**	AF139876	F: AGACCCGCGAGGTCCTCAA	91 ± 9%	15.9 ± 0.8
		R: TTGCGCTGGCCGTGTTCTG		

**H3**	AF139875	F: TACCAGAAGTCCACAGACC	95 ± 10%	16.0 ± 0.6
		R: TGGAAGCGGATGTCGGA		

**H2a**	AF139873	F: GTCGTGGCAGAGGTCTT	102 ± 15%	16.9 ± 1.2
		R: CTCCTTGTCCTTGCGGA		

**H2b**	AF139874	F: GACAACATCCGCTCCGA	101 ± 16%	17.8 ± 1.8
		R: CGAAGAGGTCGTTCACG		

^1 ^Accession number for nucleotide sequence in GenBank

^2 ^The sequences are denoted *F *for the forward primer, and *R *for the reverse primer.

^3 ^Average PCR amplification efficiencies and standard deviations are calculated from triplicate reactions performed in 3 to 5 independent experiments.

^4 ^Average C_t _values and standard deviations are calculated from triplicate reactions performed in 3 to 7 independent experiments.

**Table 3:** Gene specific primers for 5' RLM-RACE reactions

**Primers**	**5' to 3' sequence**
**H2A/outer**	CCCTCTCTTCCCTCCTT
**H2A/inner**	AGGGCAGTTAGGATGTGGTT
**H2B/outer**	ACTTCTTCGTGGACTCCTTC
**H2B/inner**	CGTGTTCCGCTTGCTGAT

**H3/outer**	GAAGAGCGAGATGATGTAGTTC
**H3/inner**	CTGGAAGCGGATGTCGGA

**H4/inner**	ATGGCGGGCTTCGTGAT
**H4/outer**	TCCCGAATCCATAGAGTGTGC
